# Thrifty Hormone Ghrelin: The Secret of Aging Muscularly

**Published:** 2020-12-31

**Authors:** Yuxiang Sun

**Affiliations:** Department of Nutrition, Texas A&M University, College Station, USA

**Keywords:** Aging, Sarcopenia, Growth hormone, Ghrelin, GHS-R

## Abstract

Sarcopenia is a debilitating muscle-wasting disease that is the major cause of frailty and disability in aging. Ghrelin (aka acylated ghrelin, AG) is a circulating peptide hormone with an unique octanoylation on Ser3. AG induces growth hormone (GH) secretion, increases food intake, and promotes adiposity and insulin resistance via its receptor, *Growth Hormone Secretagogue Receptor* (GHS-R). Unlike AG, unacylated ghrelin (UAG) is a peptide generated from the same ghrelin gene with amino acid sequence identical to AG but without the octanoylation modification, so UAG does not activate GHS-R. Intriguingly, both AG and UAG have been shown to promote differentiation and fusion of muscle C2C12 cells, regulate metabolic and mitochondrial signaling pathways in myotubes, and attenuate fasting- or denervation-induced muscle atrophy. Furthermore, it has also been shown that ghrelin gene deficiency increases vulnerability to fasting-induced muscle loss in aging mice, and AG and UAG effectively protects against muscle atrophy of aging mice. Because UAG doesn’t bind to GHS-R, it doesn’t have the undesired side-effects of elevated GH-release and increased obesity as AG. In summary, UAG has an impressive anti-atrophic effect in muscle protecting against muscle atrophy in aging, it has potential to be a unique and superior therapeutic candidate for muscle-wasting diseases such as sarcopenia.

## INTRODUCTION

Sarcopenia is a degenerative loss of skeletal muscle mass which seriously affects the independence and quality-of-life of the elderly [1,2]. The major characteristic of sarcopenia is skeletal muscle atrophy, which involves massive loss of muscle structural proteins, leading to decreased muscle mass and progressive decline of muscle function. Skeletal muscle atrophy can be triggered by aging, inactivity, malnutrition, inflammation and/or denervation. Elderly are prone to muscle loss, and sarcopenia-associated disability is a huge challenge for aging care. Given the rising aging population worldwide, it is critically important to identify preventive and therapeutic strategies to combat muscle loss during aging. Exercise is known to mitigate muscle sarcopenia by improving muscle mass and function [2,3], but most frail elderly are not physically capable of exercise, which makes muscle-boosting interventions such as exercise unsuitable for the elderly. Currently, there is no effective therapy for sarcopenia in aging.

The pathogenesis of sarcopenia is a complex one, with various metabolic mediators affecting muscle fiber size, mitochondrial function, and apoptosis of myocytes. Since Growth Hormone (GH) is known to promote muscle growth and differentiation through the GH/insulin-like growth factor (IGF) axis, gut hormone ghrelin and ghrelin mimetics have emerged as attractive potential candidates for treating sarcopenia [4]. This review will discuss the promising role of ghrelin-related peptides in muscle atrophy.

## LITERATURE REVIEW

### Ghrelin-related peptides and ghrelin receptor

Ghrelin, a 28-amino acid peptide hormone produced mainly by the stomach, has multi-faceted roles such as nutritional regulation, metabolism, and inflammation [5–9]. Classically known as the “hunger hormone,” ghrelin stimulates food intake, induces GH secretion, and promotes adiposity through its receptor, the growth hormone secretagogue receptor (GHS-R) [10,11]. Ghrelin, as a nutrient sensor, it is increased by fasting and is suppressed by feeding [12–14]. While the phenotypic impact of ghrelin deficiency in mice is very mild under hemostatic condition, its effects are much more pronounced under energy deficit. We showed that ghrelin-null mice have reduced blood glucose under 50% calorie restriction, and ghrelin ablation ameliorates the hyperglycemic phenotype of leptin-deficit ob/ob mice [15–17]. The octanoylation of ghrelin is mediated by Ghrelin O-acyltransferase (GOAT) [18,19]. Strikingly, GOAT-null mice die of hypoglycemia under 60% severe calorie restriction due to dysfunction of a GH-mediated survival mechanism [20]. Therefore, ghrelin gene is considered as “thrifty” gene because it is evolutionally conserved to help animals store energy, in order to increase their chances of survival in times of famine [21].

### Acylated ghrelin and unacylated ghrelin – two sides of the same coin

Two peptides are derived from the preproghrelin gene: acylated ghrelin (AG) is with a unique post-translational octanoylation on Ser3 by GOAT, and unacylated ghrelin (UAG) is without the octanoylation modification [19]. Octanoylation on Ser3 is required for binding to the receptor GHS-R, so AG activates GHS-R, while UAG does not [18]. Thus, originally it was thought that only AG has biological functions, so AG has often been simply referred as ghrelin; meanwhile, UAG is called inactive ghrelin. Later, a number lines of evidences have shown that UAG has biological functions as well, despite the receptor of UAG is unknown. UAG modulates lipogenic and insulin-signaling pathways in metabolically active tissues such as fat, muscle, and liver in the absence of GHS-R [22]. Also UAG has been shown to be increased after aerobic exercise [23,24], and it counteracts the metabolic response of AG, but not the neuroendocrine response of AG[25]. Even though AG and UAG are derived from the same gene, UAG is far more abundant in plasma than AG; the concentration of UAG in the circulation is more than 90%, while UAG is only ~5–10%, because AG needs to undergo additional octanoylation modification by GOAT [19]. A recent report further reveals that the ratio of AG:UAG has important biological implication in neurodegenerative diseases [26].

### Major signaling pathways in muscle metabolism and function

Sarcopenia involves muscle atrophy and mitochondrial dysfunction. Exercise has been shown to improve muscle health by improving muscle mass and function [2,3]. Exercise increases insulin-signaling regulator AKT, mammalian Target Of Rapamycin complex (mTOR), sirtuin family member SIRT3, and peroxisome-proliferator-activated receptor-g coactivator-1a (PGC-1a). The mTOR signaling has a key regulatory role in muscle cell growth, and is a critical sensor of nutritional status [27]. Exercise has been shown to attenuate age-related decline of muscle protein synthesis by activating AKT-mTOR signaling [28]. SIRT3, a member of the sirtuin family of protein deacetylases, is located in mitochondria and regulates mitochondrial function [29]. PGC-1a plays a central role in mitochondrial biogenesis [30], and exercise induces mitochondrial biogenesis by increasing PGC-1a expression and deacetylation [31,32]. It has been shown that exercise regulates SIRT3 in muscle to activate PGC-1a [33]. Adenosine monophosphate-activated protein kinase (AMPK) is a major metabolic regulator of muscle metabolism, AMPK-mediated muscle autophagy is important in maintaining integrity and mitochondrial function of muscle during prolonged fasting and myopathy in aging [34]. Since ghrelin is a potent GH secretagogue, it is considered as a candidate for treatment of sarcopenia [35]. It would be interesting to determine whether ghrelin signaling regulates these metabolic and mitochondrial pathways to elicit protective effects on muscle.

### Role of AG and UAG in myocytes

*In vitro*, it is shown that AG and UAG promote differentiation and fusion of C2C12 cells in the absence of GHS-R [36]. Interestingly, UAG exhibits both pro-anabolic and anti-anabolic effects on C2C12 myotubes exposed to cytokines [37]. More excitingly, our collaborator’s team has reported that: 1) AG and UAG act on a common, unidentified receptor to block skeletal muscle atrophy independent of GHS-R and GH/IGF-1; 2) AG and UAG inhibit skeletal muscle atrophy through PI3Kß, mTORC2, and inflammatory p38 signal pathways 3) UAG has a protective effect on muscle atrophy in mice: pharmacological administration of UAG ameliorates skeletal muscle atrophy induced by prolonged fasting or denervation [38].

Although exercise is proven to be beneficial for sarcopenia, increasing muscle anabolism by modulating metabolic regulators of AKT and mTOR, and mitochondrial regulators of SIRT3, AMPK and PGC-1a [33,39], the reality is that exercise is challenging for old people and may even be dangerous for frail elderly who suffer sarcopenia. It is exciting to see AG and UAG enhancing muscle health by regulating these signaling pathways of PI3K and mTORC2 in muscle [38] normally activated by exercise, without changing physical activity levels.

### Ghrelin gene in muscle atrophy in aging

We recently reported that old mice deficient in ghrelin are more susceptible to fasting-induced muscle atrophy, and that AG and UAG can reverse the process by promoting anabolic effects and suppressing catabolic muscle metabolism [40]. Fasting-induced muscle loss was exacerbated in old ghrelin-null mice, showing decreased expression of myogenic regulator MyoD and increased expression of protein degradation marker MuRF1, as well as altered mitochondrial function. Remarkably, AG and UAG treatments effectively increased myogenic genes and decreased degradation genes in the muscle of fasted old ghrelin-null mice [40]. We have also found that AG and UAG increase expression of insulin receptor substrates (IRS1and IRS2) and enhance activity of AMPK, and the regulating effect of UAG on these regulatory genes appears to be even more robust. Moreover, we found that AG and UAG treatments significantly increased the mitochondrial respiratory capacity of muscle C2C12 cells, which is in agreement with the protective effect observed *in vivo*. Our data together indicate that UAG ameliorates sarcopenia by activating anabolic genes and suppressing catabolic genes to increase muscle mass, and by modulating metabolic signals and mitochondrial machinery to improve muscle function [40]. Collectively, our results suggest that AG and UAG have major roles in the maintenance of aging muscle, enhancing muscle anabolism and exerting protective effects against muscle loss. AG and UAG may hold exciting promise for prevention/treatment of sarcopenia in aging.

### The therapeutic advantage of UAG in muscle

While AG has anti-atrophic effect on muscle, being a GH secretagogue and possessing orexigenic and obesogenic properties, AG also activates GH and increases obesity at the same time. In contrast, UAG possesses similar anti-atrophic effects on muscle as AG, but it doesn’t stimulate GH release nor promote obesity. High GH has been shown to potentially increase risk for cancers [41]. Since UAG does not activate GHS-R, UAG has beneficial effects on muscle but avoids the cancer and obesity risks associated with AG. In our study, it appears that UAG is more potent in regulating myogenic, insulin-signaling, and AMPK genes [40]. UAG is an effective agent protecting against muscle wasting without undesirable side-effects, which this makes it a superior therapeutic option for the prevention/treatment of muscle atrophy.

The biological effects and therapeutic implications of UAG have revealed great promise and warrant further investigation. Studies of chronological changes of UAG throughout lifespan, identification of a UAG receptor, and better understanding of the molecular mechanisms that mediate the effects of UAG in muscle will provide further insights to the understanding of UAG in pathogenesis of sarcopenia, and advance the development of therapeutic application of UAG in sarcopenia.

## CONCLUSION

**Figure 1:**
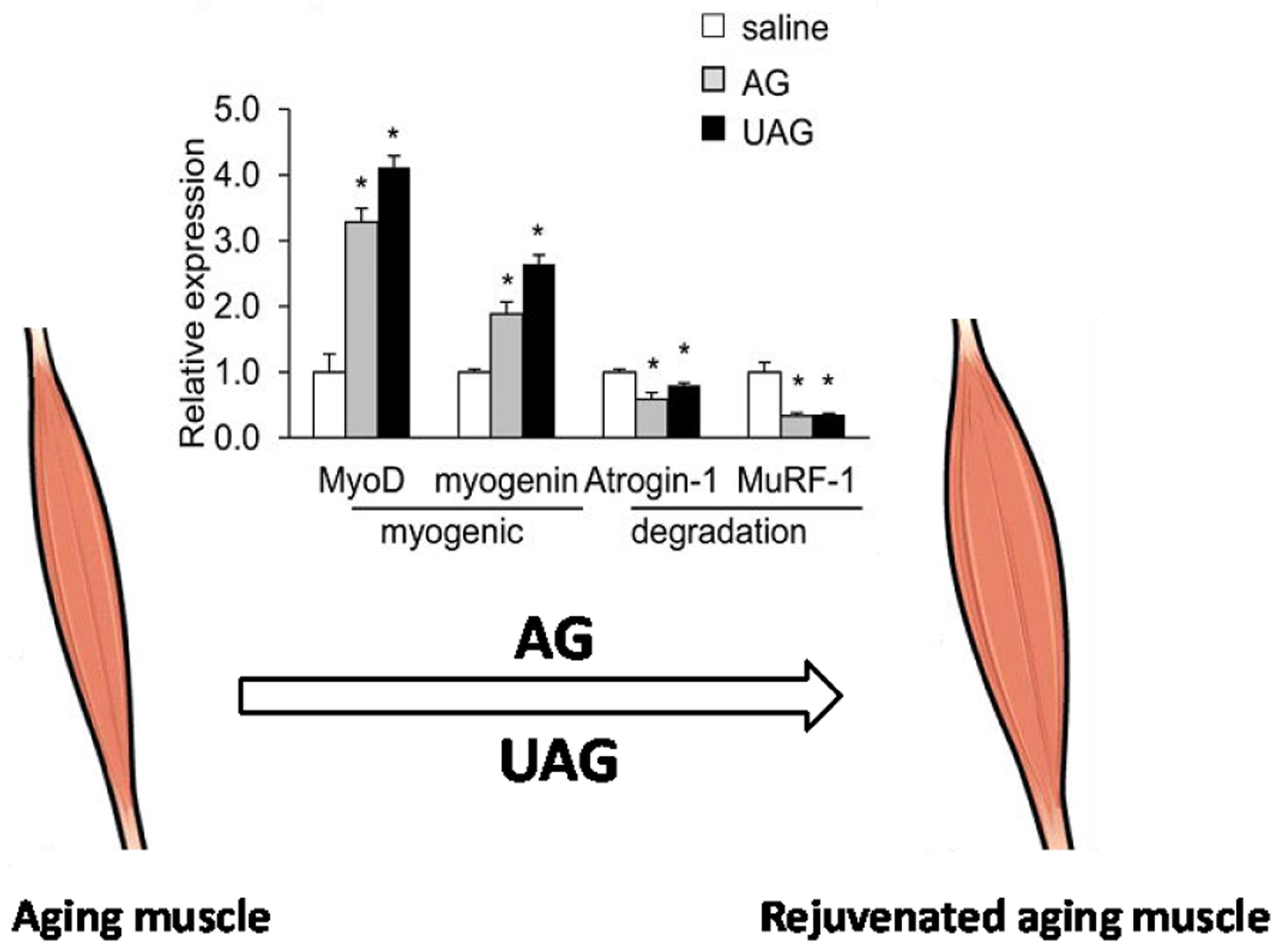
Schematic illustration of the rejuvenating effect of ghrelin-related peptides in atrophic aging muscle. AG and UAG protect against muscle atrophy in aging mice by up-regulating anabolic myogenic genes and down-regulating catabolic degradation genes. The inserted data figure is from our publication in Journal of Gerontology (2020) 75:621–630.

In summary, this review has illustrated both *in vivo* and *in vitro* evidences that ghrelin-related peptides AG and UAG have crucial roles in preserving muscle mass and improving muscle function (Figure 1).

These exciting findings suggest that ghrelin signaling has important and unique roles in maintenance of muscle health, hold great potential to be anti-sarcopenia therapy. AG and UAG trigger anti-atrophic signaling pathways directly in myocytes to protect against muscle atrophy, independent of ghrelin receptor GHS-R and the classical nourishing mechanism of GH/IGF-1 activation.

AD and UAG rejuvenate muscle without requiring physical activity, which could have tremendous benefits for frail elderly who cannot exercise. AG and UAG function as exercise mimetics to protect and nourish muscle without exercise, thus ameliorating muscle atrophy and rejuvenating aging muscle by activating exercise-like signaling pathways in muscle, offering an excellent anti-sarcopenia candidate for the elderly.

Lastly, UAG has impressive anti-atrophic effect in muscle, it increases muscle mass and improves muscle function. It is remarkable that UAG has the muscle-nourishing effects as AG, but is devoid of the potential carcinogenic and obesogenic side effects associated with AG. Thus, UAG represents a unique and more attractive therapeutic option for muscle-wasting diseases such as sarcopenia in aging. It would be beneficial to identify the receptor for UAG, and further elucidate the detailed signaling cascades mediating the effects of UAG in myocytes under normal and atrophic conditions.
